# Simultaneous development and validation of the higher education-to-work transition barriers scales in two countries: Switzerland and Togo

**DOI:** 10.1177/10384162251325549

**Published:** 2025-03-28

**Authors:** Kokou A. Atitsogbe, Jérôme Rossier

**Affiliations:** Institute of Psychology, 27213 University of Lausanne, Switzerland; Institute of Psychology, 27213 University of Lausanne, Switzerland; NCCR-LIVES, University of Lausanne, Switzerland

**Keywords:** Perceived educational and career barriers, career transitions, university students, scale development and validation, cross-cultural psychology, HEWTBS

## Abstract

This study developed and validated the Higher Education-to-Work Transition Barriers Scale (HEWTBS) to assess students’ perceived barriers when transitioning to work. Conducted in Switzerland and Togo, two culturally and economically distinct countries, it resulted in two versions: HEWTBS-CH (Switzerland) and HEWTBS-TG (Togo). The first phase involved interviews with 13 students and surveys of 617 higher education students to identify relevant items and barrier structures for each context. In the second phase, data from 452 participants were used to confirm the scale structures and analyze their relations to career decision-making difficulties, satisfaction with studies, and self-perceived employability. Despite starting with the same item pool, the selected items and structures varied between countries, showing the influence of context. The scales demonstrated good reliability and relevance in their respective contexts, with important implications for counseling, research, and future studies.

The school-to-work transition among higher education graduates seems to become more difficult in many countries as a result of increased competition in the graduate labor market (Beaumont et al., 2016). This transition is becoming longer and has reached an average of 36 months in countries such as Togo (OECD, 2016). It has been well documented that an unsuccessful transition to work usually leads to long periods of unemployment, underemployment, poor-quality employment, poverty, and increased mental health risks (Koivisto et al., 2010). To reduce these difficulties, local counseling services, states, and even international institutions are continually struggling for efficient ways to help students make a satisfactory transition to work. Unfortunately, most of the proposed employability-based solutions rarely suggested individualized interventions considering students’ perceived barriers (Agence Universitaire de la Francophonie, 2020; Beaumont et al., 2016) despite several international reports and authors highlighting the strong impact of contextual barriers (e.g., perceived lack of opportunity, constraints of the labor market) on students’ career development (Gardiner & Goedhuys, 2020; Lent et al., 2000; Swanson & Tokar, 1991a).

## Impact of perceived barriers on students’ career development

Barriers are defined as “events or conditions, either within the person or in their environment, that make career progress difficult” (Swanson & Woitke, 1997, p. 434). Albert and Luzzo (1999) described “perceived” barriers as those that individuals believe exist or might encounter, impacting their career development directly, regardless of their factual accuracy (p. 431). Perceived barriers, whether related to education or career, significantly affect academic performance, career choices, and opportunities (Atitsogbe et al., 2016; Lent et al., 2000; McWhirter et al., 2017; Swanson & Tokar, 1991a). They also influence well-being, as seen in studies where perceived skill deficits correlated with lower career decidedness (Toyokawa & DeWald, 2020) and perceived barriers were associated with reduced satisfaction in academic majors (Urbanaviciute et al., 2016). Despite these associations, the relationship between perceived barriers and perceived employability remains underexplored.

Over the years, various classifications of perceived barriers have been proposed. Initially, barriers were categorized into internal or personal barriers (e.g., lack of self-confidence) and external or environmental barriers (e.g., financial difficulties) (Creed et al., 2004; Crites, 1969; Swanson & Tokar, 1991a). Swanson and Tokar (1991a) further distinguished between social/interpersonal barriers (e.g., family and friends), attitudinal barriers (e.g., changes in interest), and interactional barriers (e.g., connections). Within Social Cognitive Career Theory, Lent et al. (2000) differentiated between objective barriers (e.g., actual financial support) and subjective barriers (e.g., perceived discrimination). They also made a distinction between distal variables, which affect individuals early in development (e.g., parental encouragement), and proximal variables, which influence the career decision-making process (e.g., lack of occupational information).

## Perceived barriers and social cognitive career theory

Social Cognitive Career Theory (SCCT; Lent et al., 1994), derived from Bandura's general social cognitive theory (Bandura, 1986), offers insights into career development, including vocational interests, choice-making, and performance. SCCT highlights the impact of contextual factors on career processes, defining barriers as “negative contextual influences” that differ from personal factors (Lent et al., 2000, p. 39). According to SCCT, perceived barriers can hinder the conversion of interests into goals and actions. For instance, students lacking financial support may be less likely to pursue their career goals (Lent, 2008). Research on perceived barriers often includes financial issues, education level, social relations, discrimination, lack of motivation or preparation, lack of psychological resources, individual characteristics, location, lack of information, or poor academic performance (Creed et al., 2004; Irvin et al., 2012; Lent et al., 2002; McWhirter et al., 2007; Migunde et al., 2012; Mohd et al., 2010). SCCT authors have noted the need for more specific measures to assess these contextual factors (Swanson & Tokar, 1991a). The theory continues to guide the development of measures and taxonomies for assessing perceived barriers in various contexts (Atitsogbe et al., 2016; McWhirter, 1997; Swanson et al., 1996).

## Assessing career-related barriers

Swanson and Tokar (1991b) have described the absence of consensus on the definition of career barriers, the necessity of developing a typology beyond the general internal–external classification, and the need to investigate the impact of several demographic variables on barriers. For this reason, they developed the Career Barriers Inventory (CBI; Swanson & Tokar, 1991b), which includes 18 scales grouped into four and five dimensions for women and men, respectively. Swanson and colleagues (1996) further developed a revised and shorter version (CBI-R) including 70 items and 13 scales: (1) Sex discrimination, (2) Lack of confidence, (3) Multiple-role conflict, (4) Conflict between children and career demands, (5) Racial discrimination, (6) Inadequate preparation, (7) Disapproval by significant others, (8) Decision-making difficulties, (9) Dissatisfaction with career, (10) Discouraged from choosing nontraditional careers, (11) Disability/health concerns, (12) Job market constraints, and (13) Difficulties with networking/socialization. The CBI-R subscales correlated relatively highly with one another, ranging from .27 (Disapproval by significant others—Disability/health concerns) to .80 (Sex discrimination—Racial discrimination), while internal consistency estimates ranged from .64 (Disapproval by significant others, Difficulties with networking/socialization) to .86 (Sex discrimination) (Swanson & Daniels, 1995; Swanson et al., 1996).

McWhirter (1997), based on Social Cognitive Career Theory (SCCT), developed the Perception of Barriers Scale (POB) to assess barriers such as discrimination and barriers to pursuing postsecondary education. The original version, consisting of 22 items, was designed for high school students, specifically Mexican-American and Euro-American. It was later revised into a 24-item version for college students to assess the extent of perceived barriers. In 2001, Luzzo and McWhirter further expanded the POB to 32 items, covering both career-related and educational barriers, with strong test-retest reliability scores between .68 and .78.

CBI-R and POB are frequently used to assess perceived career or educational barriers. Several studies have shown that barriers, as measured with these instruments, vary according to demographic variables, such as gender and sociocultural and ethnic background (e.g., Luzzo & McWhirter, 2001; Swanson et al., 1996; Watts et al., 2015). Moreover, perceived barriers have been seen to be negatively related to academic major satisfaction, vocational identity commitment, career maturity, mathematical and science self-efficacy, and (realistic) occupational aspirations and positively related to outcome expectations and career indecision (e.g., Kantamneni et al., 2018; Patton et al., 2003; Urbanaviciute et al., 2016; Watts et al., 2015). Although both measures have been used in different cultures (e.g., Cardoso & Marques, 2008; Fort & Murariu, 2018; Lindley, 2005; Soresi et al., 2012), most studies have been conducted in North America, with the POB used frequently.

## Aims of this study

This study uses a bottom-up methodology (Atitsogbe & Bernaud, 2024) to develop psychometrically adequate measures to access perceived barriers to the higher education-to-work transition in Switzerland and Togo. Indeed, these two countries are sample examples of industrialized and less industrialized countries that present notable differences regarding education conditions, employment, economic characteristics, and culture. Switzerland typically has an unemployment rate below 4%, while Togo's rate was estimated 32% for higher education graduates (OECD, 2016). Additionally, Togo faces critical study conditions that affect academic performance (Atitsogbe et al., 2016; Chitou, 2011). Cultural differences, such as Switzerland's autonomy-supporting environment versus Togo's in-group regulations, may also shape the types and number of perceived barriers. These contextual differences suggest that the nature and structure of perceived barriers within the transition from higher education to work will vary between the two countries.

## Study 1: development of the higher education-to-work transition barriers scales

Semidirected interviews were conducted among Swiss and Togolese university students. The interviews were recorded, taped into Word format, and content-analyzed to construct the pool of items as further described. Second, the pool of items was used to collect data by questionnaire among another sample of Swiss and Togolese participants for exploratory factor analyses.

### Method

#### Participants

Sample 1. The semidirected interviews were conducted with 13 volunteers, including 5 Swiss (University of Lausanne/École Polytechnique Fédérale de Lausanne) and 8 Togolese participants (University of Lomé), aged 22.80 on average (*SD *= 3.83), studying social sciences, engineering, or business.

Sample 2. The questionnaire survey included 617 university students, with 316 from Switzerland (51.22% of the sample) and 301 from Togo (48.78%). Swiss participants were aged 18 to 46 (*M *= 22.57, *SD *= 3.00) and included 32.9% men and 66.1% women. The sample comprised 23.7% third-year undergraduates and 75% master's students, with 62.3% identifying as Swiss, 27.5% as dual-nationality holders, and 8.9% as foreigners. Togolese participants were aged 17 to 40 (*M* = 21.12, SD = 2.78), including 61.8% men and 37.5% women. This sample consisted of 2.7% third-year undergraduates and 95% master's students, with 96% identifying as Togolese, 3% as dual nationality holders, and 0.3% as foreigners (0.7% did not provide such information).

#### Instruments

**Interviews Material.** Participants answered questions aimed at exploring the barriers they faced in their education and future transition to work. Sample questions included: “What are the difficulties that you have encountered during your studies at university?”, “What are your career plans after graduation?”, “Do you believe you will easily achieve your goals?”, and “What are the challenges students face when moving from university to employment?”. The interviews lasted between 11 and 34 min (*M *= 17) and were all transcribed and analyzed using a content-analysis procedure (Bardin, 1977) for item development.

**Higher Education-to-Work Transition Barriers Scale (HEWTBS).** We used the initial HEWTBS, comprising 101 items categorized into educational barriers (57 items), career barriers (39 items), and general barriers (5 items). Participants rated each item on a 7-point Likert scale from *not true at all* (1) to *very true* (7). Higher scores on the HEWTBS scales indicate a greater perception of barriers.

#### Scale development

Forty-four barriers were identified (based on identified indicators) and grouped into seven educational barriers (academic achievement, study conditions, residence to university distance, awareness and enrollment conditions, family conditions, financial needs, and motivation), eight career barriers (lack of information regarding opportunities, difficulties accessing opportunities, difficulties convincing potential employers, networking, professional profile robustness, mismatch between chosen major and desired career, and discrimination), and general barriers related to the self (e.g., self-confidence, health). Based on the 44 barriers’ indicators, a total of 101 items were generated: 57 for educational barriers, 39 for career barriers, and 5 for general barriers, all to be rated using a 7-point Likert-type scale (ranging from *not true at all* to *very true*). Six vocational psychology experts from Switzerland and Togo reviewed the items and response format for content validity, phrasing, and comprehensibility. Some items were revised, including rephrasing and reversing, based on consensus (Worthington & Whittaker, 2006).

#### Procedure

Data were collected using paper–pencil questionnaires in both Switzerland and Togo. In Switzerland, undergraduate students from the University of Lausanne, participating in a research practicum, used the snowball method to survey 10 participants each, from the French-speaking region, earning course credit for their efforts. In Togo, data was collected at the University of Lomé with assistance from a career counseling psychologist. Volunteers completed the questionnaire during their free time and were compensated with prepaid phone credit worth 2000 XOF (about 4 USD).

### Results

Preliminary analyses revealed that the structure of items was context-dependent, leading to the development of separate measures for Switzerland and Togo. Both countries’ data were tested for the Bartlett test of sphericity (*p *< .001) and Kaiser–Meyer–Olkin measure of sampling adequacy (KMO = .803 and .745, respectively), which indicated the suitability of the items for exploratory factor analysis (EFA) (Tabachnick & Fidell, 2001). EFA in each country, using Jamovi 1.1.9.0, retained items with factor loadings above .40 (Hinkin, 1998). Parallel analysis suggested 12 factors for Switzerland and 10 for Togo. However, principal axis factor analyses with oblique rotations (factors were expected to be correlated) revealed one difficult-to-interpret factor in each country, resulting in 11 factors for Switzerland and 9 for Togo, explaining 45.02% and 54.90% of the variance, respectively.

For the Swiss form of the Higher Education-to-Work Transition Barriers Scale (HEWTBS-CH), item loadings ranged from .43 to .88 (*Mdn *= .66) (Table 1). Nine items loaded on the first factor (F1_CH_), labeled Profile robustness, evaluating background and the ability to persuade employers (e.g., “I have little self-confidence that I will succeed in a job interview”). The second factor (F2_CH_), Difficulties to meet academic requirements, included eight items assessing adaptation to study tasks (e.g., “The work effort required in my studies is beyond my capabilities”). The third factor (F3_CH_), Dissatisfaction with major, included six items that assessed the match between the chosen major and the desired future career (e.g., “I'm no longer interested in my current major”). The fourth factor (F4_CH_), Unfavorable family environment, comprised five items that assessed the relationship with family and study conditions at home that may hinder academic performance (e.g., “My relationship with family/friends negatively affects my academic performance”). The fifth factor (F5_CH_), Lack of information, comprised 5 items that evaluate a lack of information about job opportunities in their field and complementary training (e.g., “I have very little information about job opportunities in my field”). The sixth factor (F6_CH_), Economic constraints, consisted of 4 items assessing difficulties regarding financial means (e.g., “I have little financial support to complete my studies”). The seventh factor (F7_CH_), Low information-seeking, consisted of 4 items evaluating the willingness to seek information regarding employment in one's field (e.g., “I don't know much about the state of the labor market in my field”). The eighth factor (F8_CH_), Lack of motivation, included four items evaluating attitude regarding studying (e.g., “I am often absent from classes”). The ninth factor (F9_CH_), Network, included four items evaluating one's social network's scope and perceived help in job-seeking (e.g., “I have a network that I can count on if I need to look for a job”). F10_CH_, Opportunities in the region, comprised 2 items assessing job opportunities in one's environment (e.g., “To find a job, I may have to leave my region”). F11_CH_, Distance home–university, contained two items assessing how unfavorable the distance between one's residence and the university is (e.g., “The distance from my residence to the university discomforts me”). Cronbach's alphas for HEWTBS-CH subscales ranged from .76 to .89 (*Mdn *= .84). Note that sample items presented in the current study have been translated from French (original items) into English.

**Table 1.** Factor loadings from exploratory factor analysis across the Swiss sample – study 1.

	Factors										
Items	F1	F2	F3	F4	F5	F6	F7	F8	F9	F10	F11
F1 : Profile robustness (α = .88)												
Ca10	**.** **812**	.021	−.030	−.022	.014	−.044	−.003	−.081	.013	.050	−.003
Ca 52	.**738**	.025	.052	.030	−.032	.090	.013	.008	−.083	.037	.031
Ca 18	.**729**	−.113	.023	.104	.009	−.069	.038	.033	.040	.008	.006
Ca 56	.**660**	.053	.030	−.067	−.015	.102	.058	.079	.014	−.003	.068
Ca 41	.**635**	−.059	.035	.154	−.016	−.093	.043	.034	.042	−.073	.043
Ca 9	.**594**	.084	−.070	−.046	.156	−.010	−.038	−.029	.139	−.024	−.004
Ca 36	.**573**	.108	−.005	−.037	.011	.001	.063	−.008	.065	−.016	−.073
Ca 48	.**566**	.087	−.025	−.080	.042	−.124	−.018	−.016	.057	−.009	−.022
Ca 15	.**543**	.023	.050	−.011	−.019	.071	−.140	.108	−.002	.029	−.011
F2 : Difficulties to meet academic requirements (α = .87)												
Ed 4	.033	.**666**	.144	.102	.006	−.069	−.025	−.145	.047	.027	.043
Ed 34	−.076	.**666**	−.018	.027	−.085	.014	.094	.178	.076	−.016	.079
Ed 25	.054	.**650**	−.045	.059	.119	.167	.039	.137	−.081	−.037	−.097
Ed 10	.062	.**642**	.065	.037	−.097	.009	.009	.155	.035	−.031	.027
Ed 8	.092	.**639**	.113	.095	.024	.033	−.055	−.141	−.020	−.005	.095
Ed 13	.012	.**619**	.023	.111	.052	−.007	.015	−.179	−.109	.016	.088
Ed 37	.073	.**555**	.027	.039	.047	.138	.084	.231	−.048	−.013	−.049
Ed 17	.099	.**433**	−.053	.073	−.002	.019	.097	.089	.041	−.043	.010
F3 : Dissatisfaction with major (α = .87)												
Ca 54	.003	−.047	.**795**	.050	−.093	−.019	.011	−.065	.049	.026	.043
Ed 16	.016	.110	.**787**	−.036	−.055	−.035	.005	.050	−.023	−.018	−.024
Ca 39	.036	−.012	.**775**	.022	.110	.123	−.045	−.020	−.075	.011	−.064
Ca 14	.017	.019	.**767**	.020	−.011	.017	≤.01	.012	.043	−.045	−.015
Ca 3	−.076	−.089	.**617**	−.089	.084	−.076	.065	.199	.049	−.006	.044
Ca 24	.001	.107	.**560**	−.007	.182	−.042	.012	.025	−.058	.027	.102
F4 : Unfavorable family environment (α = .89)												
Ed 27	.016	.016	−.026	.**883**	−.049	.029	.027	.016	−.027	−.012	−.041
Ed 7	−.027	−.004	−.030	.**831**	.030	−.083	.002	−.002	.052	−.019	.058
Ed 14	−.011	.092	.043	.**802**	.012	−.012	−.008	.003	.050	.038	−.034
Ed 31	.054	.012	.009	.**772**	.034	.136	−.013	.028	−.004	−.012	.012
Ed 44	.037	−.023	.087	.**519**	.027	.104	−.058	−.051	−.084	−.052	.078
F5 : Lack of information (α = .84)												
Ca 2	.015	−.096	.004	.021	.**729**	.015	.009	≤01	.031	.105	.116
Ed 26	−.033	.107	.040	.032	.**721**	−.037	.013	.012	.035	−.056	−.077
Ca 12	.071	−.075	.031	.042	.**691**	−.021	.090	.027	.060	.093	≤.01
Ed 18	−.011	.220	−.037	−.038	.**627**	−.057	−.014	.029	.033	−.122	−.031
Ca 37	.089	−.121	.043	−.028	.**616**	.065	.230	−.064	−.020	.033	.010
F6 : Economic constraints (α = .80)												
Ed 11	−.004	.058	.014	.145	.005	.**684**	−.029	.128	.036	.035	.067
Ed 2	−.003	.051	−.034	.044	.050	.**650**	−.039	−.092	.089	−.051	.120
Ed 15	−.035	.025	.022	.002	−.081	.**620**	−.084	−.023	.071	.025	.043
Ed 38	.019	−.004	−.025	.006	−.017	.**595**	−.016	−.132	.073	−.021	−.047
Ed 19	−.089	.019	.065	−.008	−.124	.**579**	.065	−.056	.041	.075	−.095
Ed 36	.015	−.023	.033	.028	.099	.**541**	−.087	.272	−.078	−.004	.064
F7 : Low information seeking (α = .78)												
**Ca 31**	.020	−.017	.044	.007	.044	−.106	.**764**	.003	−.003	−.071	.021
Ca 47	.078	−.044	−.033	.058	.075	.011	.**728**	.044	−.051	.103	−.027
Ca 26	−.012	.100	−.025	−.079	.047	.012	.**673**	−.032	.047	−.007	−.003
**Ca 49**	−.139	.148	.014	.007	−.019	.046	.**521**	−.084	.050	−.060	.121
F8 : Lack of motivation (α = .78)												
Ed 22	−.−.−.−.015	.063	.156	.063	.036	−.105	−.041	.**670**	.085	.011	.054
Ed 24	−.012	−.082	−.040	.021	.004	.131	−.029	.**662**	−.058	.005	.055
Ed 20	.082	.018	.089	.010	−.113	.086	.225	.**569**	.013	−.002	−.037
Ed 32	.082	.261	.088	.040	.095	−.044	−.015	.**566**	.056	.026	−.043
F9 : Network (α = .76)												
Ca 19	.064	−.019	.009	.089	.077	.073	−.020	−.004	.**734**	.070	−.034
Ca 38	.078	−.064	.035	−.053	−.043	.026	.082	−.003	.**667**	−.023	−.017
Ca 6	−.054	.014	−.009	.162	.083	.031	−.035	.042	.**633**	.041	.026
Ca 11	.038	.033	−.062	−.203	−.018	.045	−.038	.003	.**586**	−.012	.027
F10 : Opportunities in the region (α = .87)												
Ca 16	.027	.000	.008	.023	.015	.016	.003	−.018	−.036	.**880**	−.012
Ca 32	−.025	.028	−.019	−.040	−.005	−.024	−.007	.029	.059	.**869**	.028
F11 : Distance home-university (α = .83)												
Ed 39	.002	.005	.012	.042	.027	.010	←−.01	.019	−.011	−.014	.**841**
Ed 5	.027	.029	−.026	−.060	−.026	.028	.022	.001	−.003	.044	.**818**

*Note.* Ed = Educational barrier items. Ca = Carrier barrier items. *N *= 316.

For the Togolese form (HEWTBS-TG), item loadings ranged from .417 to .815 (*Mdn *= .608). (Table 2). The Economic constraints and Opportunities in the region factors comprised identical items in both countries. Five other factors were similar in both countries but were associated with different items. For instance, two items were different for Lack of information, six for Profile robustness, one for Dissatisfaction with major, one for Unfavorable family environment, and five for Difficulties to meet academic requirements. Note that the order of the factors differed between countries. Moreover, two factors were specific to the Togolese form: Unfavorable study conditions (F8_TG_) and Skills for specific job demands (F9_TG_). F8_TG_ was associated with three items assessing study conditions on campus (e.g., “Resources in my university (library, internet connection) do not allow me to study in good conditions”). F9_TG_ was associated with three items addressing external employability or demands regarding one's specific competencies (e.g., “I have specific skills that are demanded on the job market”). Cronbach's alpha for F5_TG_ was particularly low (α = .38) and ranged from .65 to .82 (*Mdn *= .75) for the other factors. As the balance between competency supply and demand has been found to be crucial in the country (Atitsogbe et al., 2021a; OECD, 2016), we decided to further keep the specific factor F5_TG_ in the Togolese model despite its low Cronbach alpha value. Further investigations will be carried out to improve this specific subscale.

**Table 2.** Factor loadings from exploratory factor analysis across the Togolese sample – study 1.

**Items**	**Factors**								
	**F1**	**F2**	**F3**	**F4**	**F5**	**F6**	**F7**	**F8**	**F9**
F1 : Economic constraints (α = .80)									
Ed15	**.** **768**	−.038	.032	−.058	−.087	.002	.033	−.044	.022
Ed 19	.**690**	−.008	.005	−.062	.044	−.007	−.080	.055	.041
Ed 36	.**642**	−.097	.018	.111	−.049	.150	.054	.030	.025
Ed 11	.**634**	.057	−.003	.008	.156	.154	.037	−.019	−.035
Ed 38	.**561**	.151	−.061	.003	.066	−.087	−.014	−.026	−.062
Ed 2	.**451**	.103	.025	.011	.073	−.151	.078	.026	−.025
F2 : Lack of information (α = .79)									
Ca 37	.067	.**687**	.075	.051	.000	.037	.022	−.020	−.008
Ed 26	−.050	.**625**	.024	−.091	.030	.112	.076	.149	.097
Ca 12	.013	.**611**	−.006	.002	.029	−.080	−.030	.003	.076
Ca 2	.031	.**531**	−.074	.169	.086	−.020	.020	−.014	−.066
Ed 18	−.038	.**526**	.017	−.084	.037	.086	−.027	.204	.042
Ca 26	−.022	.**510**	.137	.001	−.066	.152	−.008	−.162	−.068
Ca 47	−.038	.**462**	.149	.062	−.129	.037	.021	–.107	.053
F3 : Profile robustness (α = .79)									
Ca 52	.118	.070	.**630**	−.148	.050	−.049	−.011	.044	.042
Ca 41	−.002	.149	.**611**	.053	−.007	−.006	.015	−.097	.004
Ca 36	−.005	−.054	.**599**	.134	−.006	−.049	−.051	.011	−.065
Ed 40	−.003	.057	.**583**	.080	.040	.011	.038	.026	.085
Ca 56	−.011	.047	.**533**	−.005	.046	.056	.024	.057	.024
Ca 34	−.133	−.067	.**512**	.006	.006	.343	.067	.025	.008
Ca 15	.086	.042	.**417**	.109	.043	−.113	−.006	.086	.145
F4 : Dissatisfaction with major (α = .76)									
Ca 54	.050	−.014	.083	.**766**	−.037	−.004	−.022	.020	−.025
Ca 39	−.037	.047	−.058	.**734**	−.032	.070	.019	−.041	.034
Ed 16	−.006	−.040	.037	.**562**	.105	.056	.088	.134	−.029
Ca 14	−.070	.019	.048	.**533**	.040	−.021	−.086	.111	.098
Ca 3	−.140	−.005	−.080	.**429**	.082	−.009	−.104	.002	.048
F5 : Unfavorable family environment (α = .79)									
Ed 7	−.087	.009	−.031	−.062	.**753**	.039	−.005	−.059	−.028
Ed 27	.053	.109	.008	.043	.**736**	−.060	.007	−.007	.001
Ed 14	−.041	−.148	.105	.019	.**641**	.140	.105	−.021	.102
Ed 31	.295	−.016	.033	.038	.**563**	−.009	−.022	.089	−.023
F6 : Difficulties to meet academic requirements (α = .77)									
Ed 37	.064	.039	−.006	.082	−.011	.**772**	.003	.007	.041
Ed 25	.043	.098	.041	.020	.079	.**679**	.001	.021	−.074
Ed 8	.065	.044	−.050	−.018	.114	.**522**	−.095	.128	.105
F7 : Opportunities in the region (α = .75)									
Ca 32	≤.01	−.031	−.021	−.018	.024	.063	.**815**	.018	−.016
Ca 16	.015	.068	.040	←≤.01	−.004	−.124	.**735**	.001	.026
F8 : Unfavorable study conditions (α = .62)									
Ed 28	.000	.071	−.029	.073	−.030	.053	.016	.**690**	.013
Ed 35	−.039	−.080	.073	.013	−.044	.003	.061	.**563**	−.028
Ed 21	.016	.023	.026	−.011	−.041	−.048	−.025	.**510**	−.095
F9 : Specific job demands (α = .64)									
Ca 22	.069	.053	−.169	.124	.011	−.098	.130	−.009	.**630**
Ca 44	−.028	.038	.174	−.024	.039	−.005	−.108	.008	.**605**
Ca 1	−.021	−.016	.090	−.056	−.022	.147	−.009	−.048	.**596**

*Note.* Ed = Educational barrier items. Ca = Carrier barrier items. *N *= 301

Descriptive statistics for both HEWTBS-CH and HEWTBS-TG are shown in Table 3 and Table 4. For HEWTBS-CH, Skewness (*S*) ranged from −.06 (Opportunity in the region) to 1.34 (Dissatisfaction with major), and Kurtosis (*K*) ranged from −.08 (Academic requirements) to 1.73 (Dissatisfaction with major). For HEWTBS-TG, *S* values ranged from below .01 (Study conditions) to 1.21 (Dissatisfaction with major), and *K* values were between .03 (Specific job demands) and 1.11 (Dissatisfaction with major). These values fall within acceptable normality ranges (*S* values ≤ |3|; *K* values ≤ |10|; Weston & Gore, 2006). Scale scores correlated positively with one another, with significant values ranging from .13 (lack of motivation—network) to .47 (unfavorable family environment—difficulties meeting academic requirements) for HEWTBS-CH and from .13 (opportunities in the region—economic constraints) to .36 (lack of information—low profile robustness) for HEWTBS-TG.

**Table 3.** Correlations of HEWTBS subscales for the Swiss calibration sample – study 1.

	**1**	**2**	**3**	**4**	**5**	**6**	**7**	**8**	**9**	**10**	**11**
1. Profile robustness (low)	—										
2. Academic requirements	.30***	—									
3. Dissatisfaction with major	.19***	.32***	—								
4. Family environment	.24***	.47***	.22***	—							
5. Lack of information	.33***	.17**	.21***	.17**	—						
6. Economic constraints	.01	.25***	.04	.34***	−.05	—					
7. Information seeking (low)	.19***	.19***	.11	.00	.39***	−.17**	—				
8. Lack of motivation	.22***	.39***	.38***	.24***	.16**	.18**	.15**	—			
9. Network	.29***	.07	.02	.14*	.22***	.18**	.04	.13*	—		
10. Opportunities in the region	.14*	−.09	.01	−.02	.14*	.10	−.01	.03	.20***	—	
11. Distance home-university	.09	.18**	.11	.14*	.08	.19***	.08	.09	.05	.21***	—
*M*	3.02	2.75	2.16	2.24	3.38	2.59	3.95	2.89	4.27	3.92	2.99
*SD*	1.08	1.11	1.19	1.40	1.36	1.36	1.41	1.26	1.32	1.67	1.89
*S*	.45	.57	1.34	1.15	.29	.92	−.10	.60	.08	−.06	.63
*K*	−0.51	−.08	1.73	.57	−.70	.41	−.69	−.12	−.74	−.95	−.82

*Note.* HEWTBS-CH = HEWTBS Swiss Form. HEWTBS-TG = HEWTBS Togolese Form; *p < .05; **p < .01; ***p < .001.

**Table 4.** Correlations of HEWTBS subscales for the Togolese calibration sample – study 1.

**Subscales**	**1**	**2**	**3**	**4**	**5**	**6**	**7**	**8**	**9**
1. Economic constraints	—								
2. Lack of information	.16**	—							
3. Profile robustness (low)	.10	.36***	—						
4. Dissatisfaction with major	−.02	.14*	.20***	—					
5. Family environment	.34***	.16**	.21***	.08	—				
6. Academic requirements	.21***	.30***	.32***	.24***	.32***	—			
7. Opportunities in the region	.13*	.10	.08	.01	.16**	.02	—		
8. Study conditions	.05	.12*	.12*	.21***	.04	.18**	.07	—	
9. Specific job demands	.05	.22***	.24***	.15**	.14*	.15*	.10	.02	—
*M*	3.52	4.01	2.43	2.12	2.87	2.54	3.43	3.72	2.91
*SD*	1.47	1.28	1.05	1.18	1.54	1.38	1.65	1.37	1.19
*S*	.30	−.07	.71	1.21	.63	.87	.35	<.01	.54
*K*	−.77	−.23	−.12	1.11	−.37	.25	−.66	−.45	.03

*Note.* HEWTBS-TG = HEWTBS Togolese Form; *p < .05; **p < .01; ***p < .001.

## Study 2: confirmation of the structure, nomological network, and reliability

This study aimed to: (1) confirm the dimensionality and structure of the perceived barriers (HEWTBS-CH and HEWTBS-TG), (2) assess the reliabilities of the developed scales and their relations to satisfaction with studies, a set of career development variables and demographics such as age and gender —including incremental validity—, and (3) evaluate test–retest stability.

The HEWTBS scales are expected to show positive correlations with the main CDDQ scales, given that decision-making difficulties are a perceived barrier within the CBI-R(Swanson et al., 1996). However, these correlations should not be so strong as to suggest the scales are overlapping. Moreover, because perceived barriers usually relate negatively to positive career development outcomes and are associated with increased uncertainty (e.g., Atitsogbe et al., 2016; Jaensch et al., 2016; Patton et al., 2003, Urbanaviciute et al., 2016), it is reasonable to expect negative correlations between HEWTBS scales and overall satisfaction with studies and self-perceived employability. We also expect perceived barriers to be correlated with age and gender. Specifically, we expect barriers to increase with age and women to report higher levels of barriers compared to men. Additionally, we expect HEWTBS scales to predict perceived employability over CDDQ in both country samples (incremental validity) and to show stability over time (test–retest).

### Method

#### Participants

The confirmatory study included 452 university students. Switzerland: 251 participants aged 17 to 48 (*M *= 22.51; *SD *= 2.94). This group comprised 46.6% men and 54.4% women. They included 61 third-year undergraduates (24.3%) and 190 master's students (75.7%). Of the Swiss participants, 58.6% identified as Swiss, 28.3% as dual nationals, 12.7% as foreigners, and 0.4% as stateless. Togo: 201 participants aged 20 to 43 (*M *= 23.9; *SD *= 3.31). This group comprised 58.7% men and 41.3% women. They included 10 third-year undergraduates (5%) and 186 master's students (92.5%). Among them, 96.5% identified as Togolese, 0.5% as dual nationals, and 2.5% as foreigners.

To evaluate the test–retest reliability for both versions of the HEWTBS, additional data were collected from 197 (69.5% women and 30.5% men) participants in Switzerland and 235 (65.5% men and 34.5% women) in Togo, who had previously participated in the studies twelve months earlier. Most of the participants (74.1% in Switzerland and 94.9% in Togo) were master's graduates.

#### Instruments

***Higher Education-to-Work Transition Barriers Scale (HEWTBS).*** The newly developed HEWTBS-CH and HEWTBS-TG were used.

***Career Decision-making Difficulties Questionnaire (CDDQ).*** The CDDQ (Gati et al., 1996, 2000) consists of 34 items evaluating individuals’ perceived difficulties in the career decision-making process. It consists of ten subscales grouped into three main categories: Lack of readiness, which includes Rm, Ri, and Rd; Lack of information, which includes Lp, Ls, Lo, and La; and Inconsistent information, which includes Iu, Ii, and Ie. A total score represents the overall decision-making difficulties. Cronbach's alpha coefficients across the Swiss and the Togolese samples for Lack of readiness (α = .64 vs. .64), Lack of information (α = .88 vs. .93), Inconsistent information (α = .83 vs. .88), and the overall CDDQ (α = .92 vs. .93) were satisfactory.

***Satisfaction with studies.*** A single measure, “How satisfied are you with your current studies?”, designed specifically for this study, was used to assess how satisfied the university students were with pursuing their current studies. The item was rated on a 7-point Likert-type scale ranging from *not at all satisfied* (1) to *very satisfied* (5). This item was designed based on previous investigations that acknowledged the validity of a single-item approach measuring satisfaction domains (e.g., Nagy, 2002), which may be useful in the case of a long questionnaire (less time-consuming and fatigue, less expensive, and more flexible) (Samson et al., 2024).

***Self-Perceived Employability Scale.*** The student version of the Self-Perceived Employability Scale (SPES; Rothwell et al., 2008) was used to assess self-perception of participants’ ability to get a job. This 16-item measure has been reliable among Togolese university students (Atitsogbe et al., 2019). Cronbach's alpha was .85 for the Swiss and Togolese samples.

#### Procedure

Questionnaires were paper–pencil as in Study 1. For the test–retest, Swiss participants who completed the HEWTBS-CH previously (time 1) were invited by e-mail to complete the questionnaire for the second time one year later (time 2), as were Togolese participants for HEWTBS-TG. At time 2, participants were compensated with the equivalent of a 16.67 US$ gift card (Switzerland) and a 4 US$ (Togo) prepaid phone credit card, respectively.

### Results

#### Structure of HEWTBS-CH and HEWTBS-TG

Each of the factor structures of the HEWTBS derived from EFAs (Switzerland and Togo) were subjected to CFAs in Amos 26.0 with maximum likelihood estimation. Various fit indices were inspected: χ^2^ per degree of freedom (χ^2^/*df*), the comparative fit index (CFI), the Tucker–Lewis index (TLI), and the root mean square error of approximation (RMSEA). According to some psychometrical standards, a well-fitting model should exhibit χ^2^/*df* < 3, CFI and TLI ≥ .90 (Byrne, 2010), and RMSEA ≤ .08 or .05 (Hu & Bentler, 1999). A correlated-factor model and a higher-order model were tested.

Two separate correlated-factor models allowing HEWTBS-CH 11 factors and HEWTBS-TG 9 factors to correlate were assessed. The HEWTBS-CH model required one additional constraint within its Opportunity in the region factor. One of the three items of the HEWTBS-TG Study conditions factor (Ed21) that showed a particularly low loading (.19) was removed, resulting in 39 items for HEWTBS-TG. The standardized loadings from items to corresponding factors ranged from .48 to .89 (*Mdn *= .73) for HEWTBS-CH and from .30 to .79 (*Mdn *= .61) for HEWTBS-TG. The fit indices were relatively poor for HEWTBS-CH (χ^2^/*df *= 1.72, CFI = .850, TLI = .838 and RMSEA = .054) and HEWTBS-TG (χ^2^/*df *= 1.66, CFI = .811, TLI = .789 and RMSEA = .057).

Some methodologists have acknowledged that item-level models usually result in large modification indices (MIs) and poor fit (e.g., Thompson & Melancon, 1996). Given the poor fit indices of the correlated-factor model for the two scales, we moved to test subscale-level models. Separate principal component analyses (PCA) with Varimax rotation on HEWTBS-CH and HEWTBS-TG subscales (assuming uncorrelated components) yielded a two-factor solution according to parallel analysis. Consequently, the higher-order model for each instrument was designed based on PCA findings.

**Figure 1.**
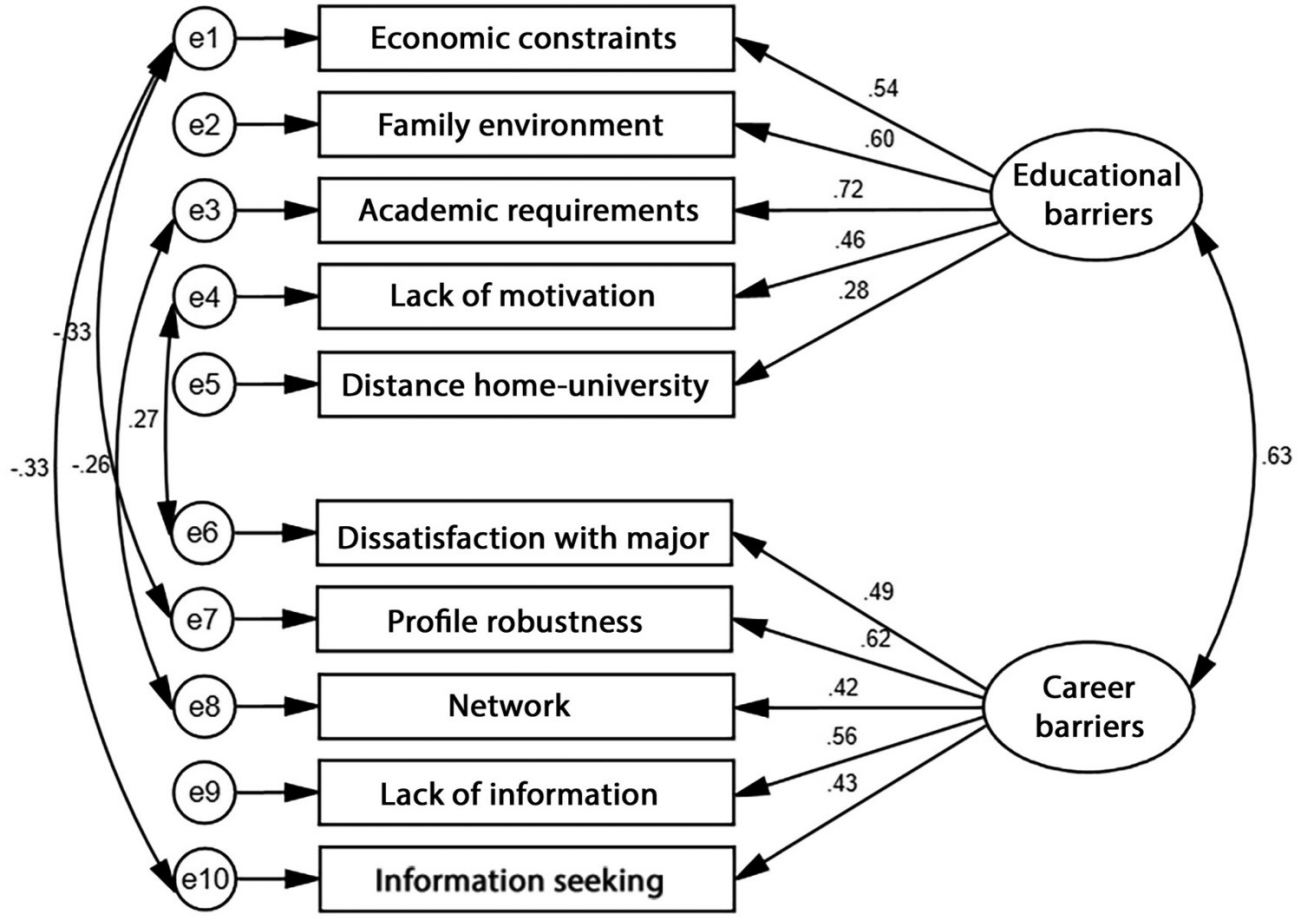
Subscale-level higher order CFA of HEWTBS-Swiss form.

For HEWTBS-CH, PCA results showed that the subscales of Economic constraints, Family environment, Academic requirements, Lack of motivation, and Distance home–university organized into a first factor labeled Educational barriers, while the subscales of Dissatisfaction with major, Profile robustness, Network, Lack of information, Information seeking, and Opportunities in the region organized into a second factor labeled Career barriers. CFA of this model allowing Educational barriers and Career barriers as higher-order factors to correlate resulted in poor fit: χ^2^/*df *= 2.83, CFI = .801, TLI = .745 and RMSEA = .086. The Opportunity in the region subscale had a very weak loading (.17) and was removed from the model. Moreover, allowing the error terms associated with MIs above 10 to correlate led to substantially improved fit: χ^2^/*df *= 1.84, CFI = .936, TLI = .903 and RMSEA = .058 (Figure 1). The standardized loadings for this final model ranged from .28 to .72 (*Mdn *= .52).

**Figure 2.**
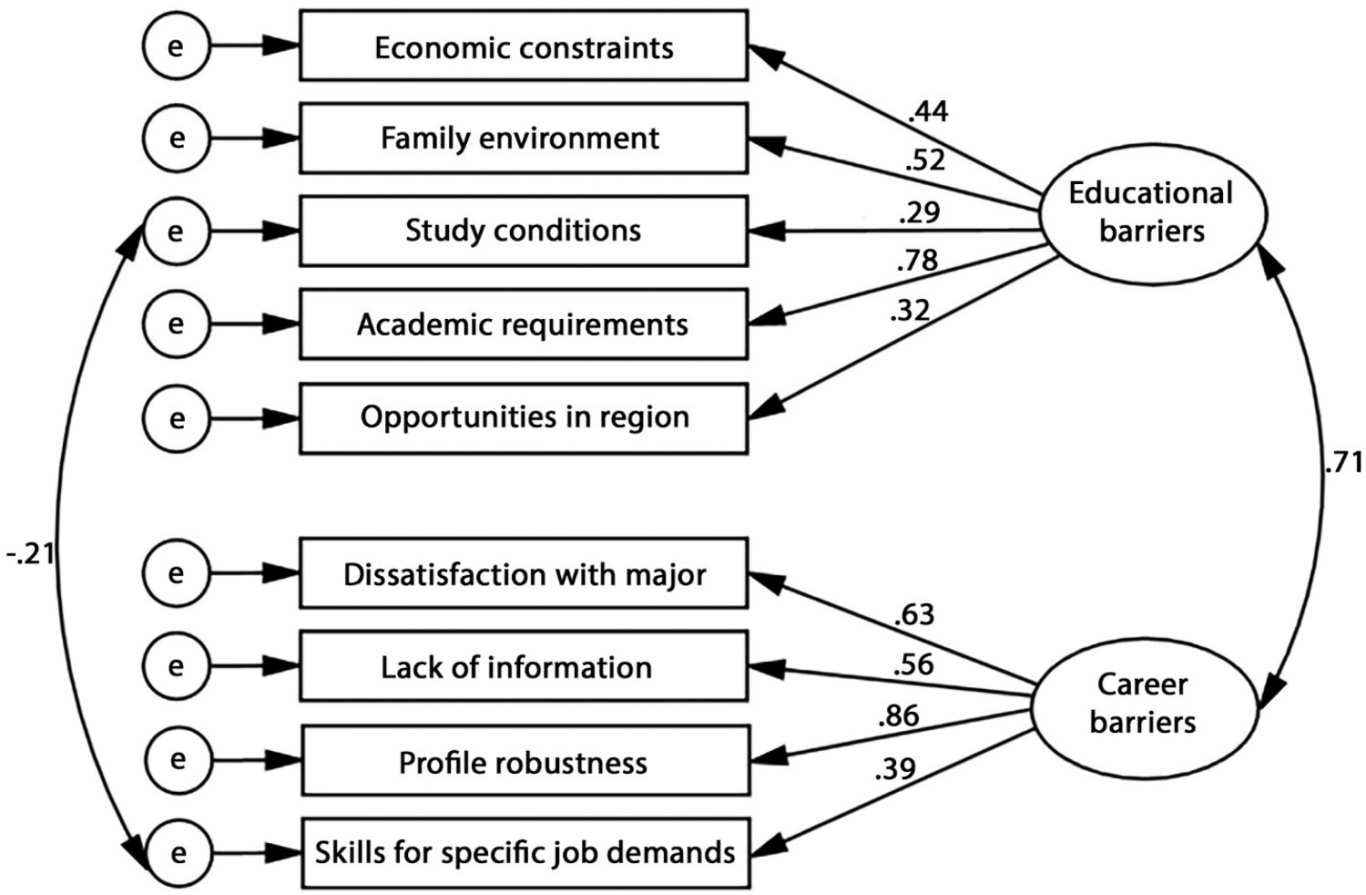
Subscale-level higher order CFA of HEWTBS-Togolese form.

For HEWTBS-TG, PCA results showed that the subscales of Economic constraints, Family environment, Study conditions, Academic requirements, and Opportunities in the region organized into a first factor named Educational barriers, while the subscales of Dissatisfaction with major, Lack of information, Profile robustness, and Skills for specific job demands organized into a second factor named Career barriers. CFA of this model allowing both Educational barriers and Career barriers as higher-order factors to correlate resulted in relatively poor fit: χ^2^/*df *= 2.14, CFI = .910, TLI = .875, and RMSEA = .075. The examination of MIs revealed that allowing the error term of Skills for specific job demands and that of Study conditions to correlate resulted in good fit: χ^2^/*df *= 1.88, CFI = .933, TLI = .903 and RMSEA = .066 (Figure 2). The standardized loadings in this final model ranged from .29 to .72 (*Mdn *= .52).

The comparison between the item-level correlated-factor model and the subscale higher-order model of both HEWTBS-CH and HEWTBS-TG revealed high differences in fit in favor of the higher-order model. Therefore, the higher-order model should be retained. The main scales (i.e., Educational barriers and Career barriers) and subscales of the final versions of both instruments showed satisfactory internal reliability coefficients ranging from .77 to .89 (*Mdn *= .87) for HEWTBS-CH (Table 5) and from .49 (2-item subscale) to .82 (*Mdn *= .76) for HEWTBS-TG (Table 6).

**Table 5.** Correlations between HEWTBS-CH and validation scales for the Swiss sample – study 2.

	**1**	**2**	**3**	**4**	**5**	**6**	**7**	**8**	**9**	**10**	**11**	**12**	**13**	**14**	**15**	**16**	**17**	**18**	**19**	**α**	**Items**
1. Economic constraints	—																			.80	6
2. Family environment	.42^c^	—																		.89	5
3. Academic requirements	.35^c^	.42^c^	—																	.87	8
4. Lack of motivation	.18^b^	.18^b^	.40^c^	—																.79	4
5. Distance home-university	.17^b^	.10	.24^c^	.20^b^	—															.77	2
**6. Educational barriers**	.71^c^	.70^c^	.80^c^	.56^c^	.39^c^	—														.89	25
7. Dissatisfaction with major	.14^a^	.25^c^	.26^c^	.37^c^	.15^a^	.35^c^	—													.89	6
8. Profile robustness	.01	.26^c^	.24^c^	.23^c^	.06	.25^c^	.35^c^	—												.89	9
9. Network	.25^c^	.21^b^	.06	.16^a^	.09	.23^c^	.16^a^	.29^c^	—											.80	4
10. Lack of information	.13^a^	.21^c^	.22^c^	.08	.13^a^	.24^c^	.24^c^	.37^c^	.20^b^	—										.82	5
11. Information seeking	−.08	.09	.22^c^	.20^b^	−.01	.14^a^	.22^c^	.21^c^	.12	.35^c^	—									.77	4
**12. Career barriers**	.13^a^	.33^c^	.32^c^	.33^c^	.14^a^	.38^c^	.65^c^	.78^c^	.50^c^	.65^c^	.54^c^	—								.89	28
13. CDDQ Lack of readiness	−.06	.15^a^	.29^c^	.29^c^	.01	.22^c^	.21^c^	.43^c^	.10	.26^c^	.36^c^	.45^c^	—							.64	10
14. CDDQ Lack of information	.10	.26^c^	.30^c^	.19^b^	.02	.29^c^	.35^c^	.57^c^	.20^b^	.56^c^	.33^c^	.65^c^	.56^c^	—						.93	12
15. CDDQ Inconsistent information	.26^c^	.39^c^	.32^c^	.29^c^	.05	.42^c^	.52^c^	.43^c^	.17^b^	.33^c^	.16^a^	.54^c^	.40^c^	.65^c^	—					.83	10
16. Overall CDDQ	.12	.32^c^	.36^c^	.28^c^	.03	.36^c^	.42^c^	.58^c^	.20	.50^c^	.34^c^	.67^c^	.74^c^	.93^c^	.80^c^	—				.92	32
17. Satisfaction with studies	−.15^a^	−.18^b^	−.34^c^	−.44^c^	−.19^b^	−.38^c^	−.56^c^	−.27^c^	−.15^a^	−.11	−.22^c^	−.43^c^	−.16^a^	−.24^c^	−.39^c^	−.31^c^	—			—	—
18. SPES	−.11	−.21^b^	−.03	−.22^c^	−.11	−.19^b^	−.30^c^	−.51^c^	−.26^c^	−.29^c^	−.13^a^	−.50^c^	−.16^a^	−.28^c^	−.24^c^	−.28^c^	.32^c^	—		.85	16
19. Age	.27^c^	.21^c^	−.05	.03	.03	.14^a^	.03	.01	−.03	−.08	−.04	−.02	−.09	−.14^a^	−.01	−.11	−.06	−.08	—	—	—
20. Sex (1 = women, 2 = men)	−.06	−.12	−.04	.13^a^	−.18^b^	−.07	.08	−.17^b^	−.05	−.12	−.08	−.07	−.01	−.08	−.05	−.06	−.10	.24^c^	−.01	—	—

*Note.* HEWTBS-CH = HEWTBS-Swiss Form. *N *= 251. CDDQ = Career Decision-Making Difficulties Questionnaire. SPES = Self-Perceived Employability Scale. ^a^ *p *< .05, ^b^ *p *< .01, ^c^ *p *< .001. Main scales are in bold. Educational barriers score is obtained through the mean of subscales 1 to 5 items. Career barriers score is obtained through the mean of subscales 7 to 11 items.

**Table 6.** Correlations between HEWTBS-TG and validation scales for the Togolese sample – study 2.

	1	2	3	4	5	6	7	8	9	10	11	12	13	14	15	16	17	18	α	Items
1. Economic constraints	—																		.79	6
2. Family environment	.36^c^	—																	.82	4
3. Study conditions	.09	.13	—																.49	2
4. Academic requirements	.36^c^	.38^c^	.26^c^	—															.72	3
5. Opportunities in the region	.14^a^	.15^a^	.19^b^	.19^b^	—														.65	2
**6. Educational barriers**	.80^c^	.72^c^	.37^c^	.67^c^	.39^c^	—													.82	17
7. Dissatisfaction with major	.04	.22^b^	.15^a^	.35^c^	.18^a^	.25^c^	—												.77	5
8. Lack of information	.19^b^	.19^b^	.03	.39^c^	.21^b^	.32^c^	.26^c^	—											.75	7
9. Profile robustness	.19^b^	.31^c^	.15^a^	.48^c^	.30^c^	.42^c^	.56^c^	.48^c^	—										.76	7
10. Skills for specific job demands	.10	.16^a^	−.12	.20^b^	−.03	.14	.35^c^	.28	.31^c^	—									.69	3
**11. Career barriers**	.19^b^	.30^c^	.09	.51^c^	.26^c^	.41^c^	.73^c^	.76^c^	.84^c^	.56^c^	—								.86	22
12. CDDQ Lack of readiness	.20^b^	.24^c^	.07	.29^c^	.23^c^	.32^c^	.30^c^	.35^c^	.50^c^	.13	.46^c^	—							.64	10
13. CDDQ Lack of information	.24^c^	.25^c^	.08	.41^c^	.17^a^	.37^c^	.35^c^	.58^c^	.58^c^	.26^c^	.64^c^	.56^c^	—						.93	12
14. CDDQ Inconsistent information	.23^b^	.31^c^	.07	.32^c^	.17^a^	.37^c^	.43^c^	.37^c^	.46^c^	.13	.50^c^	.46^c^	.73^c^	—					.88	10
15. Overall CDDQ	.26^c^	.31^c^	.09	.41^c^	.21^b^	.41^c^	.42^c^	.53^c^	.60^c^	.22^b^	.64^c^	.73^c^	.94^c^	.87^c^	—				.93	32
16. Satisfaction with studies	−.12	−.19^b^	−.10	−.20^b^	−.11	−.23^b^	−.27^c^	−.19^b^	−.29^c^	−.31^c^	−.34^c^	−.13	−.17^a^	−.14	−.17^a^	—			—	—
17. SPES	−.27^c^	−.19^b^	−.09	−.32^c^	–<.01	−.31^c^	−.23^b^	−.24^c^	−.31^c^	−.51^c^	−.40^c^	−.09	−.30^c^	−.19^b^	−.25^c^	.40^c^	—		.85	16
18. Age	.29^c^	.04	.03	.19^a^	.02	.22^c^	−.07	−.04	−.03	−.02	−.03	−.12	−.03	−.02	−.06	−.01	−.16^a^	—	—	—
19. Sex (1 = women, 2 = men)	.11	−.07	−.10	−.12	−.01	−.02	−.21^b^	−.19^b^	−.18^a^	−.15^a^	−.25^c^	−.18	−.12	−.10	−.15^a^	.02	.01	.04	—	—

*Note.* HEWTBS-TG = HEWTBS-Togolese Form. *N *= 201. CDDQ = Career Decision-Making Difficulties Questionnaire. SPES = Self-Perceived Employability Scale. ^a^ *p *< .05, ^b^ *p *< .01, ^c^ *p *< .001. Main scales are in bold. Educational barriers score is obtained through the mean of subscales 1 to 5 items. Career barriers score is obtained through the mean of subscales 7 to 10 items.

#### Nomological validity and relations with career decision-making difficulties, employability, and demographics

We evaluated the correlations between HEWTBS-CH/TG main scales, their subscales and satisfaction with studies (nomological validity) and their relations with other constructs (i.e., CDDQ, overall satisfaction with studies, perceived employability) and the demographics of age and gender.

As shown in Table 5, the HEWTBS-CH educational barriers total score correlated positively and relatively strongly with its subscales (*r *= .39—.80) as the career barriers total score did with its subscales (*r *= .54—.78). Both total scores correlated weakly (*r *= .38). Moreover, as expected, educational barriers and career barriers total scores correlated positively with CDDQ main scales of Lack of readiness (.22 vs. .45), Lack of information (.29 vs. .65), Inconsistent information (.42 vs. .54), and overall CDDQ (.36 vs. .67) and negatively with overall satisfaction with studies (−.38 vs. −.43) and self-perceived employability (−.43 vs. −.50). As observed, overall, career barriers correlated more significantly with the other career development variables than did educational barriers. Furthermore, age correlated weakly but significantly with the educational barriers total score (.14) but not with the career barriers total score, while gender correlated significantly with none of the total scores. As observed, in Switzerland, age seemed to relate to educational barriers only (older students encountered higher levels of educational barriers, and younger students encountered lower levels).

The HEWTBS-TG educational barriers total score also correlated positively and relatively strongly with its subscales (*r *= .39—.80), as the career barriers total score correlated with its subscales (*r *= .56—.84). Both total scores correlated weakly (*r *= .41). As hypothesized, educational barriers and career barriers total scores correlated positively with the CDDQ main scales of Lack of readiness (.32 vs. .46), Lack of information (.37 vs. .64), Inconsistent information (.37 vs. .50), overall CDDQ (.41 vs. .64) and negatively with overall satisfaction with studies (−.23 vs. −.34) and self-perceived employability (−.31 vs. −.40). Similarly, career barriers correlated more strongly with the validity scales than did educational barriers. Furthermore, age correlated weakly but significantly with the educational barriers total score (.22) but not with the career barriers total score, while gender correlated significantly with all career barriers and their total score (−.15 to −.25) but with none of educational barriers and their total score. As observed, similar to Switzerland, age seemed to be related to educational barriers (older students experienced higher levels of educational barriers, and younger students experienced lower levels), while in contrast, gender was related to career barriers, with Togolese women experiencing higher levels of those barriers compared to men.

#### Incremental validity

Incremental validity addresses whether a scale of interest provides additional information after considering the predictive power of previous variables (i.e., Weiner & Greene, 2007). We performed hierarchical regression analyses to assess the incremental validity of the HEWTBS-CH/TG over the overall CDDQ in predicting self-perceived employability. Age and gender were entered in a first step, followed by CDDQ in a second step. In a third step, previous steps were continued by adding HEWTBS-CH/TG main scale. After controlling for age and gender, CDDQ explained 14% of the perceived employability variance in Switzerland and 8% in Togo. These amounts of variance reached 30% in the Swiss sample and 19% in the Togolese sample, with the two main scales of HEWTBS-CH and HEWTBS-TG added to the respective models. The additional effects of HEWTBS-CH (Δ*R*^2 ^= .16, *F*(2, 244) = 28.1, *p *< .001) and HEWTBS-TG (Δ*R*^2 ^= .11, *F*(2, 194) = 13.40, *p *< .001) were particularly important. These results suggest that the HEWTBS-CH and HEWTBS-TG scales had significant incremental validity regarding the predictive power of the CDDQ on perceived employability (Table 7).

**Table 7.** Hierarchical regression predicting self-perceived employability by overall CDDQ and HEWTBS-CH/TG main scales.

	**Switzerland (HEWTBS-CH)**						**Togo (HEWTBS-TG)**					
	**Step 1**		**Step 2**		**Step 3**		**Step 1**		**Step 2**		**Step 3**	
Variables	* B(SE)*	β	* B(SE)*	β	* B(SE)*	β	* B(SE)*	β	* B(SE)*	β	* B(SE)*	β
Age	−.02(.01)	−.08	−.02(.01)	−.11	−.02(.01)	−.09	−.03(.01)	−.16*	−.03(.01)	−.17*	−.02(.01)	−.13
Sex	.27(.07)	.23***	.25(.07)	.22***	.23(.06)	.20***	.01(.08)	.005	−.03(.08)	−.03	−.09(.08)	−.08
CDDQ			−.13(.03)	−.28***	−.04(.03)	.08			−.10(.03)	−.24***	.02(.04)	.05
Edu. barriers					−.01(.04)	.02					−.08(.04)	−.14*
Car. barriers					−.38(.05)	−.55***					−.24(.06)	−.37***
*R* ^2^	.06		.14		.30		.02		.08		.19	
*ΔR* ^2^	.06		.08		.16		.02		.05		.11	

*Note.* HEWTBS-CH = HEWTBS-Swiss Form. HEWTBS-TG = HEWTBS-Togolese Form. *N_Switzerland _*= 251. *N_Togo _*= 201. Edu. barriers = Educational barriers. Car. barriers = Career barriers. * *p *< .05, **p < .01, ***p < .001.

#### Test–retest stability

The test–retest reliabilities *r*s for HEWTBS-CH ranged from medium (.56) to very strong (.82), with a median value of .68. For HEWTBS-TG, they ranged from weak (.23) to moderate (.72), with a median value of .57. Overall, these results showed satisfactory repeatability scores in the assessment of perceived barriers with HEWTBS measures in both countries (see Table 8).

**Table 8.** Test-retest reliability for HEWTBS-CH and HEWTBS-TG across the Swiss and Togolese country-samples, respectively.

**HEWTBS-CH (*N *= 197)**		**HEWTBS-TG (*N *= 235)**	
Subscales	*r*	Subscales	*r*
1. Economic constraints	.80	1. Economic constraints	.72
2. Family environment	.76	2. Family environment	.57
3. Academic requirements	.68	3. Study conditions	.43
4. Lack of motivation	.68	4. Academic requirements	.41
5. Distance home-university	.65	5. Opportunities in the region	.38
**6. Educational barriers**	.82	**6. Educational barriers**	.62
7. Dissatisfaction with major	.56	7. Dissatisfaction with major	.57
8. Profile robustness	.70	8. Lack of information	.62
9. Network	.56	9. Profile robustness	.53
10. Lack of information	.60	10. Skills for specific job demands	.23
11. Information seeking	.66	**11. Career barriers**	.60
**12. Career barriers**	.72	—	—

*Note*. HEWTBS-CH = HEWTBS-Swiss Form. HEWTBS-TG = HEWTBS-Togolese Form. *r *= Pearson correlation coefficient. Educational barriers score for HEWTBS-CH is obtained through the mean of subscales 1 to 5 while Career barriers score is obtained through the mean of subscales 7 to 11. Educational barriers score for HEWTBS-TG is obtained through the mean of subscales 1 to 5 while Career barriers score is obtained through the mean of subscales 7 to 11. Main scales are in bold.

In summary, Study 2 confirmed the multidimensional structure of perceived barriers within the higher education-to-work transition, replicating the two-dimensional model (educational and career-related barriers) found in the POB (Luzzo & McWhirter, 2001). Both the Swiss (HEWTBS-CH) and Togolese (HEWTBS-TG) versions showed high fit, indicating the stability of the barrier structure across contexts. The study supports the measures’ nomological, construct, and incremental validity, with no bias in main scales or subscales. Both instruments demonstrated differential barrier perceptions by age and gender, with age correlating with educational barriers in both countries and gender correlating with career barriers in Togo.

## General discussion

This study aimed to develop and evaluate a measure for assessing perceived barriers within the transition from university to work among students in Switzerland (HEWTBS-CH) and Togo (HEWTBS-TG). Both instruments were well-suited to their respective contexts and demonstrated overall internal consistency and validity. They assess barriers similar to existing tools, such as dissatisfaction with studies, job market constraints, networks, and financial issues (e.g., Revised POB; IPBC-R by Cardoso & Marquez, 2008; CBI-R), while also reflecting the educational versus career barriers dichotomy (Luzzo and McWhirter, 2001). However, HEWTBS-CH and HEWTBS-TG differed in several aspects, such as the structure (number of factors), the number of items per factor, and the content of several items. Furthermore, both forms had an uneven number of items per scale, leading to lower internal reliability, indicating the need for further refinement.

### Limitations and future research directions

The current study has several limitations requiring further research. Both the Swiss and Togolese samples were highly homogeneous, with nearly 90% of Swiss participants and 97% of Togolese participants being local citizens. This underrepresentation of foreigners may have obscured the detection of barriers like nationality-based discrimination, which is significant in contexts like Switzerland (Atitsogbe et al., 2020). Additionally, the smaller-than-recommended sample sizes (fewer than 300 participants) likely contributed to weaker loadings and the removal of the “Opportunity in the region” subscale in HEWTBS-CH. Both HEWTBS versions need refinement, as some subscales have few items (e.g., 2 to 9 for HEWTBS-CH and 2 to 7 for HEWTBS-TG). Enhancing the scales using item response theory could reduce larger subscales and strengthen shorter ones, improving their overall reliability (Tracey, 2010; Worthington & Whittaker, 2006).

Another limitation of the present study lies in the use of a single-item measure of satisfaction with studies. Indeed, operationalizing a construct with a single item has been subjected to methodological debates. Although it has been proven that a single-item measure of job satisfaction exhibited strong convergent and predictive validity compared to multi-item scales (e.g., Fakunmoju, 2021), such evidence is lacking in the literature regarding a single-item satisfaction with studies measure. Although such a single measure offered practical advantages within the current study (reduced survey time and respondent workload), its validity remains to be proven. Further studies would provide deeper insight into this issue.

### Implications for practice and research

This study provides a primary tool to assess perceived barriers within school-to-work transition. HEWTBS-CH and HEWTBS-TG could be used in counseling to help students navigate this transition effectively. They can be used in career interventions to help students (a) identify potential barriers, (b) assess the likelihood of encountering them, (c) develop coping strategies, and (d) seek support from their network (Lent, 2008). Counselors can use them to prioritize barriers (Atitsogbe et al., 2016) and conduct item-level analyses to better understand individual challenges (Rochat, 2019), ultimately improving students’ confidence and well-being during their transition to the workforce. Providing counseling within the school-to-work transition using such tools could be vital in contexts where job access and contextual constraints are particularly prevalent (Atitsogbe et al., 2021b; Beaumont et al., 2016; Moumoula et al., 2023).

Researchers using the HEWTBS in different contexts are advised to select the version (Swiss form vs. Togolese form) that aligns most closely with their local environment. This recommendation is based on differences in the content and number of items across the subscales of the two versions. Context-specific items, like those in the “Unfavorable study conditions” subscale of HEWTBS-TG (e.g., limited internet access), may not apply in more industrialized countries like Switzerland. Using an unsuitable version risks overlooking local nuances (Van de Vijver, 2016).
